# The developmental trajectories and modifiable factors of adolescents’ subjective well-being from late adolescence to early adulthood

**DOI:** 10.1186/s13034-025-00881-w

**Published:** 2025-03-20

**Authors:** Songli Mei, Chengbin Zheng, Leilei Liang, Marhaba Kiyum, Tongshuang Yuan, Junsong Fei, Kai Liu, Honghua Li, Xinli Lin

**Affiliations:** 1 Department of Social Medicine and Health Management, School of Public Health, Jilin University, No. 1163 Xinmin Street, Changchun, 130021 China; 2 Department of Maternal, Child and Adolescent Health, School of Public Health, Jilin University, Changchun, 130021 China

**Keywords:** Subjective well-being, Modifiable factors, Environment-wide association study, Late adolescence to early adulthood, Latent class growth model

## Abstract

**Background:**

Previous research on adolescents’ subjective well-being has not focused on the transition from late adolescence to early adulthood. Moreover, explorations of the factors influencing adolescents’ subjective well-being have mostly focused on a single level or variable. This study aimed to identify the different developmental trajectories of adolescents’ subjective well-being during this transition period and the influencing factors at different levels.

**Methods:**

This study used data from the Chinese Family Panel Studies to longitudinally track 625 adolescents aged 16–19 years in 2014 for six years. This study adopted multi-party reports and collected data on four levels of adolescents: individuals, networks, families, and communities. Using the latent class growth model to distinguish the different developmental trajectories of adolescents’ subjective well-being. Then, the environment-wide association study (EnWAS) was used to explore the factors influencing the categories of adolescents’ subjective well-being trajectories.

**Results:**

This study identified three different developmental trajectories of subjective well-being: High initial level—relatively stable group, Medium initial level—rapidly decreasing group, Low initial level—slowly rising group. The results of EnWAS confirmed that there are 15 modifiable factors associated with the trajectory classification of adolescents’ subjective well-being. The final multiple logistic regression model revealed the household book collection, tidiness of the home, desired level of education, future confidence, interpersonal relationships, social trust, sleep duration (marginal condition), all of which have significant impacts on adolescents’ subjective well-being.

**Conclusions:**

Adolescents’ subjective well-being trajectories from late adolescence to early adulthood show great heterogeneity. Adolescents’ subjective well-being may be more influenced by the personal and family environment. Targeted interventions for various modifiable factors can significantly enhance adolescents’ subjective well-being.

**Supplementary Information:**

The online version contains supplementary material available at 10.1186/s13034-025-00881-w.

## Introduction

Subjective well-being is defined as an individual’s cognitive and affective assessment of his or her own life, and is one of the important measures of individual mental health [1]. It is worth noting that previous research on adolescent mental health has mostly focused on negative psychological states, like depression, anxiety, and social fears. However, with the rise and development of positive psychology, more and more scholars call for more attention to be paid to people’s positive psychological qualities [2]. Among them, subjective well-being, as a positive psychological state, is considered an important part of positive psychology. Subjective well-being not only has great intrinsic value to individuals, but also brings positive spillover benefits [3]. For the adolescent population, higher subjective well-being is often associated with greater academic achievement, adaptability, and positive behavioral conduct [4, 5]. Additionally, subjective well-being is closely associated to psychological symptoms like depression and anxiety in adolescents. Higher levels of subjective well-being can help alleviate negative emotional experiences to some extent, thereby better maintaining mental health.

Adolescence is a period of rapid physical, cognitive, and psychosocial development of the individual [6]. At the same time, adolescence is a sensitive period. The rapid changes in physical, psychological, and cognitive thinking that adolescents experience during this period pose serious challenges to their daily lives and may lead to a decline in subjective well-being [7, 8]. In particular, from late adolescence to early adulthood, when most adolescents complete their education and gradually enter society, the ensuing changes in social roles and life pressures are very likely to affect adolescents’ subjective well-being.

### Different developmental trajectories of subjective well-being from late adolescence to early adulthood

Previous studies have explored the trajectory of adolescents’ subjective well-being to some extent during adolescence, but these studies have not reached the same conclusions [7, 9]. For example, a previous study of German adolescents found that their subjective well-being showed a decreasing trend from 5th to 12th grade [7]. However, another investigation of the well-being of Finnish adolescents in mid- and late adolescence showed the opposite result. This study found that adolescents’ life satisfaction showed an upward trend during the longitudinal follow-up period [9]. Meanwhile, a longitudinal study tracking the growth of college students in the Chinese capital also found that their levels of subjective well-being rise over time during their college years [10]. Moreover, these previous studies have also paid less attention to the trajectory of subjective well-being during this particular transition period—from late adolescence to early adulthood. However, this period is not only a brief period of transition from adolescence to adulthood, but also a critical time for further exploration of love, work, and worldview [11]. Therefore, understanding the trajectory of young people’s subjective well-being during this transition period will not only help to better maintain individuals’ mental health, but will also enhance their quality of life and social adaptability later in life.

The inconsistency in the results of adolescents’ developmental trajectories of subjective well-being in previous studies may indicate that there may be great heterogeneity in the developmental trajectories themselves. As adolescents in this period tend to have higher susceptibility to stress, emotional and mental health problems, etc., this tends to lead to diverse trends in the subjective well-being of different adolescents. In recent years, a growing number of studies have gradually confirmed this as well [12, 13]. Among them, longitudinal evidence of more than 20,000 young Australian people showed that their subjective well-being can be divided into three profiles (low, moderate to high) and three transitional patterns: stable, partially-stable, and unstable [13]. Another large cohort study of American high school seniors also confirmed the existence of different development trajectories of well-being: steady-high, high-decreasing, low-increasing and steady-low [12]. Therefore, based on these previous findings, the present study also hypothesizes that adolescents’ subjective well-being from late adolescence to early adulthood may also show different developmental trajectories.

Considering the above shortcomings in current research, this study will focus on adolescents from late adolescence to early adulthood, and explore the different developmental trajectories of Chinese adolescents’ subjective well-being during this period through the latent class growth model (LCGM). LCGM has unique advantages in analyzing the developmental trajectories of subjective well-being and the heterogeneity among trajectories. It is able to attribute individuals to different subcategories through their initial levels and developmental rates, and can systematically describe the characteristics and developmental trends of each subcategory of subjective well-being [14].

### **Modifiable factors influencing the classification of adolescents’ subjective well-being trajectories**

Factors influencing subjective well-being range from genetic to environmental [1]. A previous review article concluded that subjective well-being has a moderate heritability [1]. Approximately 30–40% of individual differences in subjective well-being can be attributed to genetic factors. This means that most of the remaining individual differences in subjective well-being can be explained by environmental factors. Therefore, adolescent subjective well-being can be further improved through many modifiable factors.

There is a strong sensitivity of adolescent subjective well-being to changes in the individual’s surrounding environment [15]. Based on Bronfenbrenner’s Bioecological Theory [16], it is inferred that adolescents’ subjective well-being may be influenced by the microsystem, mesosystem, exosystem, macrosystem, and chronosystem. Within this theoretical framework, the adolescent’s living environment is not isolated, but is influenced by various surrounding environments [17]. For example, a survey of Greek adolescents found that specific personality traits (e.g. neuroticism, extraversion), family (e.g. perceived relationship with mother), and school factors (e.g. learning climate) were significant predictors of adolescents’ subjective well-being [18]. Secondly, in terms of the potential cumulative benefits at the community level, urban green space can significantly affect people’s subjective well-being [19]. At the macro level, national income per capita, income inequality and corruption are all correlated to some extent with people’s subjective well-being [20, 21, 22]. All of the above survey analyses indicate that, in addition to individual factors, environmental factors at different levels, such as family and community, are closely related to subjective well-being. From the perspective of the Bioecological Theory, this also highlights the potential importance of factors at difference levels (e.g. individual, family, community) in influencing adolescents’ emotional experiences and subjective well-being.

In the digital age, the impact of the network environment on the physical and mental health of young people is gaining attention. Based on Bronfenbrenner’s Bioecological Theory, Navarro and Tudge proposed the Neo-ecological Theory in 2023, which highlights the influence of technological and virtual environments on adolescents’ psychological and behavioral development [23]. Nowadays, for adolescents who are in close contact with the Internet on a daily basis, the network environment and behavior are factors that cannot be ignored in influencing adolescents’ psychology. For example, a study on seeking social support through Facebook found that when adolescents perceive social support from Facebook, it can reduce their depressive symptoms and improve their mental well-being [24]. However, when this social support is not perceived, it can actually exacerbate depressive feelings. Despite this, recent studies seem to disagree with this perspective, arguing that the internet is not the primary factor influencing adolescent mental health [25, 26]. In an article published last year, it was noted that over time, social media has been found to be one of the least influential factors on adolescents’ mental health, with more influence coming from their home and school environments [25]. Therefore, in the current study, there is no consistent conclusion on whether the network environment and network use have a significant impact on the psychology of adolescents. This study will incorporate adolescents’ Internet use behavior and combine other levels of environmental variables to comprehensively explore their relationship with subjective well-being.

Previous research on the factors influencing adolescents’ subjective well-being has mostly focused on a single or limited number of variables, with less exploration of different environmental levels and multivariate influencing factors. In order to remedy the shortcomings of previous studies, the present study will adopt environment-wide association studies (EnWAS) to comprehensively explore the factors that influence different trajectories of adolescents’ subjective well-being from late adolescence to early adulthood. Drawing on the ideas and principles of Genome Wide Association Study (GWAS), EnWAS is a non-targeted, agnostic, hypothesis-generating approach designed to broadly explore multiple environmental factors associated with a particular health outcome [27]. EnWAS is able to comprehensively test the relationship between various environmental exposures and adolescents’ subjective well-being in an unbiased manner [28]. This method goes beyond the limitations of traditional epidemiological studies that only focus on a single exposure and outcome. For example, in an EnWAS of cognitive functioning in Chinese adolescents, 43 modifiable factors were found to be associated with cognitive performance [29]. Moreover, EnWAS has also been applied in other areas, such as autism [28], childhood obesity [30], and blood pressure [31]. Therefore, from the perspective of Bronfenbrenner’s Bioecological Theory and the Neo-ecological Theory, this study conducts an extensive literature review to summarize the variables identified in previous research that significantly impact adolescents’ subjective well-being. Using EnWAS, it explores the factors at various levels that impact the classification of subjective well-being trajectories from late adolescence to early adulthood.

### Current study

Firstly, this study focuses on the specific transition period—from late adolescence to early adulthood. Second, based on the above discussion, considering that there may be great heterogeneity in the development trajectory of adolescent subjective well-being, LCGM will be used in this study to analyze the differences in the developmental trajectories of subjective well-being across individuals. Third, to compensate for the fact that previous studies have mostly focused on the influence of a single factor on subjective well-being, this study will use the form of multi-party reports (individuals, family members, interviewers, community leaders) to collect data from four levels: individual, network, family, and community. Using multiple informants will help reduce bias from a single source of information while enabling the collection of more comprehensive data. Then, the EnWAS will be conducted to explore the associations between variables at different levels and the classification of adolescents’ subjective well-being trajectories.

In summary, the overall aim of this study is to analyze the different developmental trajectories of adolescents’ subjective well-being from late adolescence to early adulthood using LCGM. Then, based on EnWAS, the study examines the potential influence of factors from various environmental levels on the classification of these developmental trajectories during this transitional period.

## Methods

### Participants

The sample population of this study was from the China Family Panel Studies (CFPS). Survey data from CFPS 2014,2018, and 2020 were used in this study. CFPS is a national, large-scale, multidisciplinary social tracking survey project. It aims to reflect China’s social, economic, demographic, and educational changes by tracking and collecting data at three levels: individual, household, and community. The CFPS baseline survey began in 2010 and has been followed by longitudinal tracking surveys every two years since then.

CFPS adopts the multi-stage probability proportional sampling method. The survey covers 25 provinces/municipalities/autonomous regions in China, representing 95% of China’s population. The respondents include all family members in the household. The CFPS includes four main types of questionnaires: adult questionnaire, children questionnaire, family questionnaire, and community questionnaire.

This study focused on a specific population—adolescents from late adolescence to early adulthood. The present study used multi-party report data from the adult questionnaire, family questionnaire, and community questionnaire. Focus on analyzing the different developmental trajectories of subjective well-being in adolescents during this transition period and their potential influencing factors. For this purpose, this study included adolescents aged 16–19 years in 2014 and conducted an in-depth analysis combined with subsequent follow-up data for up to 6 years. After excluding invalid questionnaires such as lost subjects and missing key variables, 625 participants were finally included in the data analysis. This study obtained informed consent from all participants and their guardians. The CFPS project was approved by the Biomedical Ethics Committee of Peking University (Review Approval Number: IRB00001052-14010).

### Measurements

#### Subjective well-being

In this study, participants’ subjective well-being was measured by the question “How happy do you feel?” Answers were scored on a scale of 0–10, with higher scores indicating higher subjective well-being of the participants [32].

#### Modifiable/intervenable factors

To comprehensively explore the factors that influence the classification of subjective well-being development trajectories in adolescents, this study included variables from four levels: individual level, network level, family level and community level (for specific descriptions of the variables, see Table S1).

The individual level variables mainly included variables such as smoking, drinking, sleep duration, duration of exercise per week, future confidence, social trust, and interpersonal relationships, and other variables.

The network level variables mainly included variables such as whether to go online, frequency of using the Internet to study, frequency of using the Internet for work, frequency of using the Internet for socializing, frequency of using the Internet for entertainment, and other variables.

The family level variables mainly included variables such as expenditure on culture and entertainment, expenditure on social donations, family size, household book collection, neighborhood relationships, tidiness of the home, family net income per capita, and other variables.

The community level variables mainly included variables such as whether there are high-polluting enterprises nearby, the number of full-time social workers, community economic status, the proportion of poor families, the proportion of the floating population, members’ spiritual outlook, and other variables.

#### Covariates

Previous studies have shown that gender and age are also important factors affecting adolescents’ subjective well-being. And both are immutable variables. Therefore, this study included gender and age as covariates in the analysis.

### Data analysis

This study used IBM SPSS Statistics 24.0 software and Mplus7.4 for data processing and statistical analysis. Firstly, descriptive analysis was conducted on the personal information of the participants. Next, LCGM was constructed using Mplus 7.4 to distinguish the different developmental trajectories of subjective well-being in adolescents from late adolescence to early adulthood. In determining the optimal number of classes in the LCGM, the study comprehensively employed AIC, BIC, aBIC, LMR, BLRT, and Entropy indicators. Smaller values for AIC, BIC, and aBIC indicate a better model fit. Significant LMR and BLRT values (*P* < 0.05) suggest that the model with k classes fits better than the model with (k − 1) classes. Entropy reflects the classification accuracy of the model, with values closer to 1 indicating higher classification precision.

Secondly, in order to comprehensively explore the intervenable factors that influence the classification of subjective well-being trajectories in adolescents, this study adopted EnWAS, including modifiable factors from four different levels. For individual-level and network-level factors, this study used the CFPS personal database for analysis. For family-level exposure factors, this study was based on the household codes of individuals in the personal database, matched one by one in the CFPS family database, and processed and analyzed the data. In addition, for community-level exposure factors, this study was based on village codes in the personal database, matched one by one in the CFPS village database, and conducted subsequent analysis. Then, controlling for covariates, based on the EnWAS, the present study constructed 46 independent multivariate logistic regression models to explore whether each modifiable factor was associated with different classifications of adolescents’ subjective well-being trajectories.

Finally, this study incorporated significant variables from 46 independent regression models into the final overall multivariate logistic regression model to further explore the important modifiable factors influencing the classification of subjective well-being trajectories in adolescents.

## Results

### Descriptive characteristics of participants

Table 1 presents the descriptive statistics of the participants in this study across three stages (T1, T2, T3). In this study, female participants accounted for 50.1%, with a nearly balanced male-to-female ratio. The average age of participants increased from 17.48 years at T1 to 23.48 years at T3. The proportion of participants living in urban/town areas rose from 46.2% at T1 to 55.7% at T3. The percentage of participants who were attending school decreased from 78.9% at T1 to 17.6% at T3. The smoking rate increased from 8.2% at T1 to 20.0% at T3, while the drinking rate decreased from 5.1% at T1 to 4.3% at T3. Additionally, the rate of mobile phone use saw a significant rise, increasing from 82.7% at T1 to 99.2% at T2. The proportion of participants taking afternoon naps also increased, rising from 51.8% at T1 to 56.0% at T3. Over time, adolescents’ subjective well-being scores decreased, from 8.10 points at T1 to 7.49 points at T3.

**Table 1:** Descriptive information of participants in the three waves

Variable	T1	T2	T3
	N (%)/M ± SD	N (%)/M ± SD	N (%)/M ± SD
Gender (Female)	313 (50.1)	313 (50.1)	313 (50.1)
Age	17.48 ± 1.16	21.47 ± 1.18	23.48 ± 1.17
Residential area			
Urban/Town	289 (46.2)	348 (55.7)	348 (55.7)
Rural	336 (53.8)	277 (44.3)	277 (44.3)
Currently attending school			
Yes	493 (78.9)	285 (45.6)	110 (17.6)
No	132 (21.1)	340 (54.4)	515 (82.4)
Smoking			
Yes	51 (8.2)	113 (18.1)	125 (20.0)
No	574 (91.8)	512 (81.9)	500 (80.0)
Drinking			
Yes	32 (5.1)	32 (5.1)	27 (4.3)
No	593 (94.9)	593 (94.9)	598 (95.7)
Using mobile phones			
Yes	517 (82.7)	620 (99.2)	–
No	108 (17.3)	5 (0.8)	–
Afternoon napping			
Yes	324 (51.8)	340 (54.4)	350 (56.0)
No	301 (48.2)	285 (45.6)	275 (44.0)
Subjective well-being	8.10 ± 1.66	7.62 ± 1.74	7.49 ± 1.78

### Different developmental trajectories of subjective well-being

In order to better explore the change trajectories of subjective well-being in different participants from late adolescence to early adulthood, this study constructed the LCGM. Through the LCGM, different subgroups of the changing trajectories of participants’ subjective well-being can be identified. The results show that as the number of classes increases, both AIC and aBIC decrease, indicating an improvement in model fit. Additionally, the 3-class model showed significant LRM and BLRT (*P* < 0.05), suggesting that the 3-class model fits better than the 2-class model. However, the fit of the 4-class model was not significantly better than that of the 3-class model (LMR: *P* > 0.05). Moreover, the 3-class model had the highest entropy (0.819), reflecting the highest classification accuracy. Therefore, considering all these factors, the 3-class model was chosen as the optimal model for the change trajectory of subjective well-being in this study (see Table 2).

**Table 2:** Fit indicators of the latent class model of subjective well-being

Model	Log likelihood	AIC	BIC	aBIC	LMR	BLRT	Entropy
1-class	− 3683.836	7377.672	7399.860	7383.986	–	–	–
2-class	− 3602.074	7220.148	7255.650	7230.252	0.0025	0.0000	0.715
3-class	− 3561.052	7144.105	7192.920	7157.996	0.0153	0.0000	0.819
4-class	− 3542.975	7113.951	7176.079	7131.631	0.0608	0.0000	0.750
5-class	− 3534.637	7103.274	7178.716	7124.744	0.5704	0.0000	0.748

*AIC*  akaike information criterion, *BIC* Bayesian Information Criterion, *aBIC*  the sample-adjusted Bayesian information criterion, *LMR*  the Lo-Mendell-Rubin, *BLRT*  bootstrapped likelihood ratio test

**Fig. 1:**
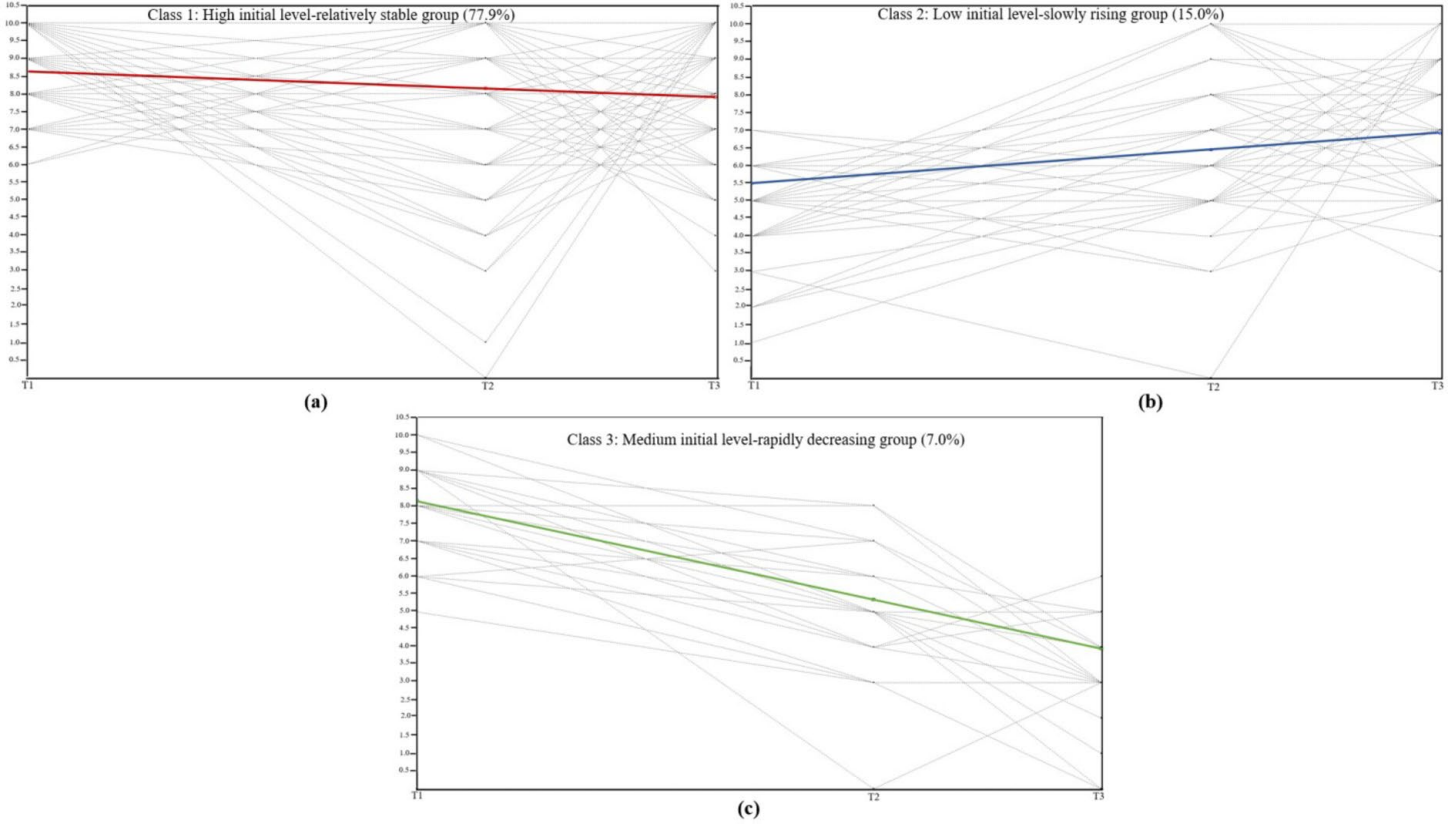
Three developmental trajectories of adolescents’ subjective well-being from late adolescence to early adulthood

The trajectory of adolescents’ subjective well-being from late adolescence to early adulthood was shown in Fig. 1. According to the characteristics in Fig. 1(a), the initial level of subjective well-being in this group of adolescents was the highest, and compared to the other two groups, the change in subjective well-being in this group was the smallest. Therefore, this group was named the “high initial level—relatively stable group”. According to the characteristics in Fig. 1(b), this group had the lowest initial level of subjective well-being, but it increased in the later stages (still lower than “high initial level—relatively stable group”), so it was named the “low initial level–slowly rising group”. Finally, according to the characteristics in Fig. 1c, the subjective well-being of the adolescents in this group had a medium initial level, but decreased rapidly in the later stages, so this group was named “medium initial level—rapidly decreasing group”. The initial levels (intercept) and slope parameters of the three change trajectories of subjective well-being were presented in Table 3.

**Table 3:** Relevant parameters of three developmental trajectory in subjective well-being

	Parameter	High initial level—relatively stable group	Low initial level—slowly rising group	Medium initial level—rapidly decreasing group
Means	I	8.643***	5.501***	8.122***
	S	-0.248***	0.473***	-1.387***

***P < 0.001, I = Intercept, S = Slope

### Modifiable factors associated with the developmental trajectories of subjective well-being

In order to explore the modifiable factors associated with the developmental trajectory of subjective well-being, this study first constructed 46 independent multiple logistic regression models. Within these models, this study found that a total of 15 modifiable variables were associated with different developmental trajectories of adolescents’ subjective well-being from late adolescence to early adulthood. Among them, individual-level modifiable variables mainly included: at school, smoking, sleep duration, desired level of education, risk of mental disorders, future confidence, interpersonal relationships, social trust, self- assessment /interviewer-assessment health status. Family-level environmental exposure factors mainly included: family social status, household book collection, neighborhood relationships, and tidiness of the home. Community-level environmental exposure factors mainly included: the proportion of poor families. The above modifiable factors were all related to the classification of the developmental trajectory of subjective well-being in transitional adolescents (see Table S2).

### Identifying key factors influencing of subjective well-being trajectory categories

The 15 significant variables in the independent Logistic regression model were included in the final multiple logistic regression model to further explore the factors influencing the trajectory classification of adolescents’ subjective well-being (see Table 4). Compared with the “moderate initial level—rapidly decreasing group”, adolescents with a higher desired level of education were more likely to belong to the “high initial level—relatively stable group”. Similarly, compared to the “low initial level—slow increase group”, adolescents who had greater confidence in the future, better interpersonal relationships, or higher levels of social trust were more likely to belong to the “high initial level—relatively stable group”. At the family level, the study found that household book collection and tidiness of the home were also protective factors for adolescents’ subjective well-being. Compared with the “low initial level—slow increase group”, when the household book collection was larger or the family was tidier, the trajectory of adolescents’ subjective well-being was more likely to belong to the “high initial level—relatively stable group”. In addition, the findings showed that sleep duration was a marginal condition influencing the trajectory of subjective well-being (*P* = 0.05).

**Table 4:** The multiple logistic regression model for different trajectory categories of adolescent subjective wellbeing

Variable	Class 2 (low initial level-slowly rising group)		Class 3 (medium initial level-rapidly decreasing group)	
	B	OR (95%CI)	B	OR (95%CI)
Currently attending school				
No	Ref		Ref	
Yes	0.110	1.117 (0.491, 2.540)	-0.673	0.510 (0.192, 1.356)
Smoking				
No	Ref		Ref	
Yes	0.589	1.802 (0.713, 4.555)	0.079	1.082 (0.350, 3.346)
Sleep duration	0.130	1.139 (0.957, 1.355)	0.214	1.239* (1.000,** 1.535)**
Desired level of education	− 0.007	0.993 (0.750, 1.315)	− 0.350	0.705* (0.505,** 0.983)**
Risk of mental disorders	0.041	1.042 (0.959, 1.132)	− 0.037	0.964 (0.861, 1.078)
Future confidence	− 0.329	0.719* (0.524,** 0.988)**	− 0.035	0.965 (0.627, 1.486)
Interpersonal relationships	− 0.343	0.710*** (0.599,** 0.842)**	− 0.040	0.961 (0.772, 1.196)
Social trust	− 0.130	0.878*** (0.837,** 0.921)**	− 0.032	0.968 (0.912, 1.027)
Self-assessment health status	− 0.195	0.823 (0.613, 1.106)	0.065	1.067 (0.728, 1.564)
Interviewer-assessment health status	0.000	1.000 (0.752, 1.331)	0.075	1.078 (0.743, 1.564)
Family social status	− 0.280	0.756 (0.529, 1.080)	− 0.326	0.721 (0.470, 1.109)
Household book collection	− 0.207	0.813** (0.705,** 0.937)**	0.026	1.026 (0.861, 1.224)
Neighborhood Relationships	− 0.307	0.735 (0.531, 1.018)	− 0.014	0.987 (0.649, 1.499)
Tidiness of the home	− 0.259	0.772* (0.622,** 0.957)**	− 0.206	0.814 (0.629, 1.052)
The proportion of poor families	1.900	6.687 (0.701, 63.808)	0.807	2.241 (0.136, 36.965)

Class 1 (High initial level—relatively stable group) as the reference group, *P<0.05, **P<0.01, ***P<0.001, The bolded OR values indicate statistical significance.

## Discussion

### Developmental trajectories of adolescents’ subjective well-being from late adolescence to early adulthood

Through the LCGM, it was found that there were three subgroups of adolescents’ subjective well-being developmental trajectories from late adolescence to early adulthood: high initial level—relatively stable group, medium initial level—rapidly decreasing group, low initial level—slowly rising group. In this study, 77.9% of adolescents’ subjective well-being trajectories belonged to the “high initial level–relatively stable group”. The period from late adolescence to early adulthood is an important time for the development of self-differentiation [33]. Self-differentiation during this period can also further maintain individual subjective well-being through adolescents’ self-construction [34]. At the same time, from late adolescence to early adulthood, the emotional regulation ability of adolescents continues to enhance, which is conducive to reducing the risk of individual mental health problems and better maintaining their subjective well-being [35]. In addition, family bonds and collectivism are valued in China [36]. Although adolescents in late adolescence may face great pressure regarding further education [37], the warmth and care from their families, as well as the supportive and collaborative atmosphere in schools, provide sufficient emotional and practical support. This helps them better cope with daily stress and maintain a high level of subjective well-being. Therefore, to a certain extent, the above discussion explains the “high initial level—relatively stable” development trajectory of subjective well-being in adolescents during this transitional period.

In the present study, over a six-year longitudinal follow-up period (from late adolescence to early adulthood), the adolescent subjective well-being trajectory of the “medium initial level—rapidly decreasing group” showed a rapid downward trend. During this transition period, adolescents tend to experience many major life events and transitions, like completing school, entering the workforce, and starting married life [11]. According to the Stress-coping Model [38], the stress and mishandling of these events may cause mental problems such as anxiety and depression, which in turn may lead to a decline in young people’s subjective well-being. In addition, individuals in adolescence tend to be confident about the future. However, according to the Multiple Discrepancies Theory [39] (Expectation and reality), when they enter adulthood, real-life challenges and difficulties may cause them to develop a huge psychological gap, which in turn may affect their subjective well-being. All of the above reasons may lead to varying degrees of decline in subjective well-being among adolescents from late adolescence to early adulthood.

In this study, it was also found that some adolescents’ subjective well-being trajectories showed a trend of “low initial level—slowly rising”. The presence of multiple potential stressors, such as the sensitivity of puberty, changes in responsibilities, and rapid hormonal changes, may cause adolescents to become more anxious and depressed, reducing their subjective well-being in life [40]. At the same time, adolescents in the later stages of puberty often face greater academic pressures [41]. Frequent exams and heavy study workloads typically drain a significant amount of their energy. This can place a heavy psychological burden on them, and if not managed properly, may further lead to a relatively lower well-being in both their life and studies [42]. However, the transition from late adolescence to early adulthood is also a pivotal period for the development of self-concept clarity. With age, cognitive maturity, and increased social interactions, self-concept clarity increases in adolescents from late adolescence to early adulthood [43]. Higher self-concept clarity tends to imply that adolescents during this period have higher levels of self-consistency and self-confidence, which can go some way to reducing adolescents’ internalization problems and thus increase their subjective well-being [44]. Therefore, the discussion above helps to explain why some adolescents in this transitional phase exhibit a trajectory of subjective well-being characterized by a “low initial level—slowly rising”.

### Modifiable factors influencing the classification of adolescents’ subjective well-being trajectories

The second part of this study aimed to explore intervenable factors that influence the classification of adolescents’ subjective well-being trajectories using EnWAS. After controlling for covariates, out of 46 modifiable factors, the study found 15 factors to be significantly associated with the classification of subjective well-being trajectories. In the final multivariate logistic regression model, modifiable factors and environmental exposures from the individual and family levels were found to be significant factors influencing adolescents’ subjective well-being from late adolescence to early adulthood.

There is no doubt that personal personality, psychological characteristics, and behavior patterns are among the most direct and important factors affecting adolescents’ subjective well-being. Secondly, the family is the closest environment to adolescents, which will have a very important influence on the development of adolescents’ psychological and behavioral habits [45]. This has been confirmed in many previous studies on adolescent mental health and subjective well-being [46]. However, for the community environment, community factors may affect the psychology and behavior of adolescents more indirectly through, for example, the family and school environments. A cross-level study on adolescents also confirmed that structural community adversity can affect adolescents’ depressive symptoms through the chain mediation of community social resources and family social resources [47]. In addition, network-level variables were found to have no significant impact on the trajectory of adolescents’ subjective well-being in this study. This result is similar to the findings of several studies in recent years. A study from last year showed that, compared to factors in the real family and school environments, social media use is one of the least influential factors on adolescent mental health [25]. A meta-analysis also found no evidence that screen media leads to suicidal thoughts or other mental health symptoms in adolescents [48]. Additionally, Orben and Przybylski, through analysis of three large-scale social datasets, similarly confirmed that the use of digital technology has only a small association with adolescents’ subjective well-being, which may be insufficient to drive relevant policy changes [26]. However, the lack of significance of network-level variables in this study may also be due to the limited number of network-level variables included and the single measurement method used. The inclusion of more network-level variables simultaneously (multiple measurement approach) could be considered in future studies for a more comprehensive analysis.

Specifically, adolescents with a higher desired level of education or greater confidence for the future are more likely to be in the “high initial level—relatively stable group”. Expectation itself is a positive state of mind, representing one’s confidence and pursuit of the future [49]. Adolescents, who expect to be more educated, tend to have clear goals for their future and are willing to work hard for it. This positive mindset, the sense of fulfilment in pursuing goals, and the sense of achievement that comes from achieving them tend to keep adolescents’ subjective well-being high. In addition, according to the Broaden-and-build Theory of Positive Emotions [50], future confidence, as an embodiment of positive emotions, tends to make teenagers adopt more positive coping styles in the process of facing pressure and challenges, and produce positive emotional experiences. Therefore, positive psychological qualities such as future confidence also have a positive impact on adolescents’ subjective well-being [51]. On the one hand, schools and parents should help adolescents develop a correct outlook on learning and encourage them to set clear goals. On the other hand, providing more guidance on educational planning and career development for adolescents will help enhance their confidence in the future, thereby improving adolescents’ subjective well-being during this transitional period.

In this study, it was found that interpersonal relationships were also an important factor affecting the trajectory classification of transitional adolescents’ subjective well-being. Adolescents with good interpersonal relationships were more likely to be in the “high initial level—relatively stable group” of subjective well-being. This result further confirmed the previous findings. For example, a study based on nationwide survey data in Germany showed that the quantity and quality (as well as different dimensions) of interpersonal relationships are of great significance in predicting the subjective well-being of adolescents [52]. Moreover, social support provided by interpersonal relationships is one of the most relevant factors for well-being [53]. According to the Social Support Theory, it can be inferred that social support from different groups can enhance adolescents’ confidence and psychological resilience, thereby increasing their subjective well-being [54]. In daily life, adolescents should actively maintain communication and interaction with peers to establish stable interpersonal relationships. At the same time, families, schools, and society can provide a positive interactive environment and cultivate social skills, thus further improving adolescents’ interpersonal relationships and enhancing their well-being levels.

Another important factor influencing the classification of subjective well-being trajectories of adolescents in transition was social trust. Similar to interpersonal relationships, adolescents with higher social trust were likely to perform better in terms of subjective well-being. A previous meta-analysis of the relationship between 137 personality traits and subjective well-being found that trust is one of the traits most closely related to subjective well-being [55]. Moreover, social trust may also indirectly affect an individual’s health and well-being through social networks and support [56]. Social trust is also a core concept of social capital. According to the Social Capital Theory, higher social trust may provide adolescents with more social capital, which in turn enhances their subjective well-being [57]. Therefore, creating a social atmosphere of honesty and trustworthiness and increasing the level of trust between people is conducive to maintaining adolescents’ subjective well-being. Moreover, encouraging adolescents to actively participate in social activities to develop their collective consciousness and cooperative spirit also helps strengthen trust and understanding among individuals.

At the family level, this study found that adolescents from families with more books and tidier homes are more likely to be in the “high initial level—relatively stable group” of subjective well-being. This suggests that family environment and atmosphere are important factors influencing adolescents’ subjective well-being. Family culture plays an important role in people’s psychological and personality development [58]. The number of books in a family also reflects family object cultural capital to a certain extent [59]. In an article on the intellectual-cultural orientation of family culture, it was argued that, as inferred from the “Estrin Paradox”, the cultural resources that an individual possesses, in addition to economic resources, often predict his or her well-being [58]. Furthermore, a greater amount of family cultural capital tends to foster adolescents’ ability to think independently and solve problems, which helps them maintain stronger psychological resilience in the face of challenges, thereby enhancing their subjective well-being. On the other hand, there are similarities between this study’s findings on home tidiness and those of previous studies. For example, a survey of Japanese adults found that less indoor cleaning was associated with people’s lower subjective well-being and poorer self-rated health [60]. From the environmental psychology perspective, a clean home environment is more likely to keep adolescents in a happy mood and positive emotional experiences, which in turn enhances their subjective well-being in daily life. Additionally, the cleanliness of the home is directly related to the sense of safety and stability among its members [61]. A clean home environment can provide adolescents with a greater sense of security, which helps reduce their stress and anxiety, ultimately improving their overall well-being. Therefore, every family should stock as many books as possible that are suitable for adolescents, cultivate good reading habits, and create a strong family cultural atmosphere. At the same time, maintaining a clean and organized home environment provides adolescents with a comfortable and relaxed living space. These measures will, to some extent, improve adolescents’ daily mood and enhance their level of well-being.

This is despite the fact that in the final multiple logistic regression model the effect of the proportion of poor families in the community on the trajectory of adolescents’ subjective well-being was found to be insignificant (this indicator was significant in the independent regression model). This may be due to the multiple influences of variables from different levels on adolescents’ subjective well-being trajectories in the final model. In the final regression model, proximal factors from the family and individual levels may have a stronger explanatory power for changes in adolescents’ subjective well-being, which in turn may have made the effect of the “the proportion of poor families in the community” on subjective well-being changes insignificant. Still, we cannot ignore the important role of the community environment in the development of adolescents’ physical and mental health [62]. The proportion of poor families in the community can reflect the economic status of the community to some extent. Communities with better economic conditions can provide more quality resources and support for adolescents, like educational resources and living service facilities. An article in *Science* has already pointed out that improved economic conditions of a community promotes long-term improvement in people’s subjective well-being [63]. Therefore, measures aimed at promoting community economic development and optimizing infrastructure may also improve adolescents’ subjective well-being. Based on community economic development, providing residents with good healthcare and education services, convenient living facilities, and rich cultural and recreational activities can greatly improve the living experiences of adolescents and, consequently, increase their subjective well-being.

## Strengths and limitations

Based on data from a national survey, this study used the LCGM to identify different trajectories of Chinese adolescents’ subjective well-being from late adolescence to early adulthood by longitudinal tracking 16–19 year olds for up to 6 years. Moreover, the important factors influencing the categories of adolescents’ subjective well-being trajectories were explored in an all-round way at four levels: individual, network, family, and community, using the EnWAS. This provides an important practical reference for further enhancing Chinese adolescents’ subjective well-being and developing specific and targeted interventions.

However, some limitations do exist in this study. First of all, the data used in this study came from questionnaires, which may have some subjective bias. The inclusion of objective measure indicators could be considered in future studies. Second, in the process of exploring the influencing factors of adolescents’ subjective well-being using EnWAS, the interaction between the variables and their non-linear relationship with the dependent variable were not taken into account. In future studies, we will continue to explore the latest data analysis methods to further examine the potential interactions among these variables and their impact on adolescents’ subjective well-being. Third, this study only includes a limited number of network-level variables. Future research will further consider exploring a broader range of digital behavior indicators for a more comprehensive analysis.

## Conclusions

This study identified three developmental trajectories of adolescents’ subjective well-being from late adolescence to early adulthood: high initial level—relatively stable group, medium initial level—rapidly decreasing group, and low initial level—slowly rising group. Based on the EnWAS and the final multiple logistic regression model, the present study found that the desired level of education, future confidence, interpersonal relationships, social trust, sleep duration (marginal condition), household book collection, tidiness of the home are all very important modifiable factors related to adolescents’ subjective well-being. Improving various modifiable exposures can effectively enhance the subjective well-being of adolescents from late adolescence to early adulthood.

## Electronic supplementary material

Below is the link to the electronic supplementary material.

Supplementary Material 1

## Data Availability

The datasets used and analyzed during the current study are available from the corresponding author on reasonable request.
