# Supplementary material for: The developmental trajectories and modifiable factors of adolescents’ subjective well-being from late adolescence to early adulthood
Source: Child Adolesc Psychiatry Ment Health. 2025 Mar 20;19:21. doi: 10.1186/s13034-025-00881-w (PMC11927348; doi:10.1186/s13034-025-00881-w)
Supplement: Supplementary file 1 — Supplementary Material 1 [file 13034_2025_881_MOESM1_ESM.docx]

**Supplementary material:**

Table S1 Variables and details at the individual, network, family, and community levels.

| **Variable** | **Reporter** | **Questionnaire type** |
| --- | --- | --- |
| **1, Individual level** |  |  |
| (1) Currently attending school (yes/no) | Self-reporting | Individual questionnaire |
| (2) Smoking (yes/no) | Self-reporting | Individual questionnaire |
| (3) Drinking (yes/no) | Self-reporting | Individual questionnaire |
| (4) Afternoon napping (yes/no) | Self-reporting | Individual questionnaire |
| (5) Sleep duration (continuous variable) | Self-reporting | Individual questionnaire |
| (6) Duration of exercise per week (continuous variable) | Self-reporting | Individual questionnaire |
| (7) BMI (continuous variable) | Self-reporting | Individual questionnaire |
| (8) Number of dinners with family per week (continuous variable) | Self-reporting | Individual questionnaire |
| (9) Total number of books read last year (continuous variable) | Self-reporting | Individual questionnaire |
| (10) What is your desired level of education? (1-8 points) | Self-reporting | Individual questionnaire |
| (11) Risk of mental disorders (Kessler 6 Rating Scale) (0-24 points) | Self-reporting | Individual questionnaire |
| (12) How confident are you about your future? (1-5 points) | Self-reporting | Individual questionnaire |
| (13) Interpersonal relationships (0-10 points) | Self-reporting | Individual questionnaire |
| (14) Unfair experiences experienced/witnessed (0-7 points) | Self-reporting | Individual questionnaire |
| (15) Social trust (0-50 points) | Self-reporting | Individual questionnaire |
| (16) Self-assessment health status (1-5 points) | Self-reporting | Individual questionnaire |
| (17) Interviewer-assessment health status (1-7 points) | Interviewer report | Individual questionnaire |
| (18) Level of attire neatness (1-7 points) | Interviewer report | Individual questionnaire |
| (19) The respondent’s appearance (1-7 points) | Interviewer report | Individual questionnaire |
| **2, Network level** |  |  |
| (1) Surf the Internet (yes/no) | Self-reporting | Individual questionnaire |
| (2) Frequency of using the internet for learning (0-6 points) | Self-reporting | Individual questionnaire |
| (3) Frequency of using the Internet for working (0-6 points) | Self-reporting | Individual questionnaire |
| (4) Frequency of using the Internet for socializing (0-6 points) | Self-reporting | Individual questionnaire |
| (5) Frequency of using the Internet for entertainment (0-6 points) | Self-reporting | Individual questionnaire |
| (6) Frequency of using the Internet for business activities (e g. shopping) (0-6 points) | Self-reporting | Individual questionnaire |
| **3, Family level** |  |  |
| (1) Family social status (1-5 points) | Self-reporting | Individual questionnaire |
| (2) What was your household’s expenditure on cultural and entertainment activities last year? (continuous variable) | Family member | Family questionnaire |
| (3) What was your household’s expenditure on travel last year? (continuous variable) | Family member | Family questionnaire |
| (4) What was your household’s social donation expenditure last year? (continuous variable) | Family member | Family questionnaire |
| (5) Family size (continuous variable) | Family member | Family questionnaire |
| (6) Family net income per capita (continuous variable) | Family member | Family questionnaire |
| (7) Household book collection (continuous variable) | Family member | Family questionnaire |
| (8) Relatives’ contacts and liaison (1-4 points) | Family member | Family questionnaire |
| (9) Neighborhood Relationships (1-5 points) | Family member | Family questionnaire |
| (10) Tidiness of the home (1-7 points) | Interviewer report | Family questionnaire |
| (11) Residential crowding degree (1-7 points) | Interviewer report | Family questionnaire |
| **4, Community level** |  |  |
| (1) Community nature (neighborhood committee/village committee) | Village/Residential Cadres | Village/Residential Questionnaire |
| (2) High-polluting enterprises nearby (yes/no) | Village/Residential Cadres | Village/Residential Questionnaire |
| (3) Number of full-time social workers (continuous variable) | Village/Residential Cadres | Village/Residential Questionnaire |
| (4) The proportion of the floating population (continuous variable) | Village/Residential Cadres | Village/Residential Questionnaire |
| (5) The proportion of poor families (continuous variable) | Village/Residential Cadres | Village/Residential Questionnaire |
| (6) Community economic status (1-7 points) | Interviewer report | Village/Residential Questionnaire |
| (7) Tidiness of roads (1-7 points) | Interviewer report | Village/Residential Questionnaire |
| (8) Members’ spiritual outlook (1-7 points) | Interviewer report | Village/Residential Questionnaire |
| (9) Homogeneity of residents (1-7 points) | Interviewer report | Village/Residential Questionnaire |
| (10) Community housing congestion level (1-7 points) | Interviewer report | Village/Residential Questionnaire |

Table S2 Independent multiple logistic regression models for different trajectory categories of adolescents' subjective well-being

| Variable |  | Class 2 (Low initial level － slowly rising group) | |  | Class 3 (Medium initial level － rapidly decreasing group) | |  |
| --- | --- | --- | --- | --- | --- | --- | --- |
|  |  | B | OR (95%CI) |  | B | OR (95%CI) |  |
| **1, Individual level** | |  |  |  |  |  |  |
| (1) Currently attending school | No | ref |  |  | ref |  |  |
|  | Yes | －0.708 | **0.493** (0.293, 0.828)** |  | －1.446 | **0.235*** (0.119, 0.468)** |  |
| (2) Smoking | No | ref |  |  | ref |  |  |
|  | Yes | 1.098 | **2.997** (1.469, 6.115)** |  | 0.867 | 2.379 (0.867, 6.523) |  |
| (3) Drinking | No | ref |  |  | ref |  |  |
|  | Yes | －0.098 | 0.907 (0.330, 2.493) |  | 0.252 | 1.286 (0.360, 4.590) |  |
| (4) Afternoon napping | No | ref |  |  | ref |  |  |
|  | Yes | －0.183 | 0.833 (0.534, 1.298) |  | －0.183 | 0.833 (0.448, 1.549) |  |
| (5) Sleep duration |  | 0.127 | 1.136 (0.974, 1.325) |  | 0.248 | **1.281* (1.049, 1.565)** |  |
| (6) Duration of exercise per week | | －0.015 | 0.985 (0.946, 1.025) |  | －0.007 | 0.993 (0.943, 1.046) |  |
| (7) BMI | | －0.089 | 0.915 (0.836, 1.002) |  | 0.069 | 1.071 (0.969, 1.185) |  |
| (8) Number of dinners with family per week | | －0.011 | 0.989 (0.910, 1.075) |  | 0.101 | 1.107 (0.967, 1.267) |  |
| (9) Total number of books read last year | | 0.005 | 1.005(0.995, 1.015) |  | －0.013 | 0.987(0.953, 1.023) |  |
| (10) Desired level of education | | －0.237 | **0.789*(0.656, 0.949)** |  | －0.543 | **0.581***(0.454, 0.743)** |  |
| (11) Risk of mental disorders | | 0.095 | **1.099**(1.031, 1.173)** |  | －0.033 | 0.968(0.860, 1.089) |  |
| (12) Future confidence | | －0.686 | **0.504***(0.384, 0.661)** |  | －0.121 | 0.886(0.590, 1331) |  |
| (13) Interpersonal relationships | | －0.521 | **0.594***(0.511, 0.691)** |  | －0.070 | 0.933(0.757, 1.148) |  |
| (14) Unfair experiences experienced/witnessed | | －0.090 | 0.914(0.803, 1.040) |  | 0.016 | 1.017(0.867, 1.192) |  |
| (15) Social trust | | －0.162 | **0.850***(0.815, 0.887)** |  | －0.044 | 0.957(0.907, 1.010) |  |
| (16) Self-assessment health status | | －0.433 | **0.649***(0.508, 0.828)** |  | 0.027 | 1.027(0.716, 1.474) |  |
| (17) Interviewer-assessment health status | | －0.247 | **0.781*(0.622, 0.981)** |  | －0.053 | 0.949(0.680, 1.323) |  |
| (18) Tidiness of clothing | | －0.166 | 0.847(0.672, 1.067) |  | －0.011 | 0.989(0.708, 1.382) |  |
| (19) Appearance | | －0.200 | 0.819(0.660, 1.015) |  | －0.007 | 0.993(0.720, 1.371) |  |
| **2, Network level** | | |  |  |  |  |  |
| (1) Surf the internet | | No | ref |  |  | ref |  |
|  | | Yes | 0.036 | 1.037(0.591, 1.821) |  | －0.132 | 0.876(0.416, 1.844) |
| (2) Frequency of using the Internet for learning | | | －0.073 | 0.929(0.839, 1.030) |  | －0.076 | 0.927(0.803, 1.071) |
| (3) Frequency of using the Internet for working | | | －0.165 | 0.848(0.717, 1.004) |  | 0.017 | 1.017(0.852, 1.214) |
| (4) Frequency of using the Internet for socializing | | | －0.006 | 0.994(0.906, 1.090) |  | 0.009 | 1.009(0.886, 1.150) |
| (5) Frequency of using the Internet for entertainment | | | 0.011 | 1.011(0.919, 1.112) |  | －0.016 | 0.984(0.864, 1.122) |
| (6) Frequency of using the Internet for business activities (e g. shopping) | | | 0.049 | 1.050(0.900, 1.225) |  | 0.045 | 1.046(0.839, 1.305) |
| **3, Family level** | |  |  |  |  |  |  |
| (1) Family social status | | －0.496 | **0.609***(0.455, 0.816)** |  | －0.292 | 0.747(0.495, 1.126) |  |
| (1) Expenditure on culture and entertainment | | －0.041 | 0.960(0.752, 1.224) |  | －0.096 | 0.909(0.604, 1.367) |  |
| (2) Expenditure on travel | | 0.041 | 1.042(0.857, 1.267) |  | －0.490 | 0.613(0.226, 1.660) |  |
| (3) Expenditure on social donations | | －0.103 | 0.902(0.685, 1.188) |  | －0.245 | 0.783(0.463, 1.323) |  |
| (4) Family size | | －0.012 | 0.988(0.851, 1.147) |  | 0.009 | 1.009(0.820, 1.241) |  |
| (5) Family net income per capita | | －0.139 | 0.870(0.643, 1.178) |  | －0.209 | 0.811(0.493, 1.337) |  |
| (6) Household book collection | | －0.196 | **0.822***(0.733, 0.922)** |  | －0.079 | 0.924(0.790, 1.080) |  |
| (7) Relatives’ contacts and liaison | | －0.060 | 0.942(0.719, 1.234) |  | －0.242 | 0.785(0.555, 1.113) |  |
| (8) Neighborhood Relationships | | －0.356 | **0.700*(0.529, 0.927)** |  | －0.058 | 0.944(0.633, 1.408) |  |
| (9) Tidiness of the home | | －0.287 | **0.750***(0.634, 0.888)** |  | －0.219 | 0.803(0.636, 1.015) |  |
| (10) Residential crowding degree | | －0.057 | 0.944(0.816, 1.093) |  | －0.120 | 0.887(0.723, 1.088) |  |
| **4, Community level** | |  |  |  |  |  |  |
| (1) Community Nature | Neighborhood | ref |  |  | ref |  |  |
|  | Village | －0.261 | 0.770(0.460, 1.288) |  | －0.323 | 0.724(0.359, 1.458) |  |
| (2) High-polluting enterprises nearby | No | ref |  |  | ref |  |  |
|  | Yes | －0.293 | 0.746(0.417, 1.334) |  | 0.328 | 1.389(0.690, 2.796) |  |
| (3) Number of full-time social workers | | －0.040 | 0.960(0.906, 1.018) |  | －0.015 | 0.985(0.919, 1.057) |  |
| (4) The proportion of the floating population | | －0.954 | 0.385(0.089, 1.667) |  | －2.306 | 0.100(0.008, 1.267) |  |
| (5) The proportion of poor families | | 1.867 | **6.468*(1.079, 38.751)** |  | 1.675 | 5.338(0.429, 66.359) |  |
| (6) Community economic status | | －0.070 | 0.933(0.794, 1.095) |  | －0.176 | 0.839(0.672, 1.047) |  |
| (7) Tidiness of roads | | －0.060 | 0.942(0.813, 1.091) |  | －0.077 | 0.926(0.754, 1.136) |  |
| (8) Members’ spiritual outlook | | －0.044 | 0.957(0.812, 1.129) |  | －0.134 | 0.875(0.697, 1.098) |  |
| (9) Homogeneity of residents | | －0.023 | 0.977(0.829, 1.151) |  | 0.096 | 1.101(0.871, 1.393) |  |
| (10) Community housing congestion level | | －0.116 | 0.891(0.768, 1.032) |  | －0.053 | 0.948(0.770, 1.167) |  |

Note: **P*＜0.05, ***P*＜0.01, ****P*＜0.001.
